# Adsorption Behavior of Organoarsenicals over MnFe_2_O_4_-Graphene Hybrid Nanocomposite: The Role of Organoarsenic Chemical Structures

**DOI:** 10.3390/ma16247636

**Published:** 2023-12-14

**Authors:** Binxian Gu, Haijie Zhang, Meng Ye, Ting Zhou, Jianjian Yi, Qingsong Hu

**Affiliations:** 1 College of Environmental Science and Engineering, Yangzhou University, 196 West Huayang Road, Yangzhou 225127, China; bxgu@yzu.edu.cn (B.G.); mz120211210@stu.yzu.edu.cn (H.Z.); mz120221245@stu.yzu.edu.cn (M.Y.); mz120221241@stu.yzu.edu.cn (T.Z.); jjyi@yzu.edu.cn (J.Y.); 2 Jiangsu Collaborative Innovation Center for Solid Organic Waste Resource Utilization, Nanjing 210095, China

**Keywords:** MnFe_2_O_4_, graphene, adsorption, p-arsanilic acid, roxarsone

## Abstract

As a kind of emerging contaminant, organoarsenic compounds have drawn wide concern because of their considerable solubilities in water, and the highly toxic inorganic arsenic species formed during their biotic and abiotic degradation in the natural environment. Thus, the effective removal and studying of the adsorption mechanism of organoarsenic compounds are of significant urgency. In this work, MnFe_2_O_4_ and MnFe_2_O_4_/graphene were prepared through a facile solvothermal method. From the results of the Transmission Electron Microscope (TEM) characterization, it can be found that MnFe_2_O_4_ nanoparticles were uniformly distributed on the surface of the graphene. And the specific surface area of the MnFe_2_O_4_/graphene was about 146.39 m^2^ g^−1^, much higher than that of the MnFe_2_O_4_ (86.15 m^2^ g^−1^). The interactions between organoarsenic compounds and adsorbents were conducted to study their adsorption behavior and mechanism. The maximum adsorption capacities of MnFe_2_O_4_/graphene towards p-arsanilic acid (p-ASA) and roxarsone (ROX) were calculated to be 22.75 and 30.59 mg g^−1^. Additionally, the ionic strength, negative ions, and humus were introduced to investigate the adsorption performance of organoarsenic compounds. Electrostatic adsorption and surface complexation are the primary adsorption mechanisms on account of X-ray photoelectron spectroscopy (XPS) and the Fourier-transform infrared spectroscopy (FT-IR) analysis. This research extends the knowledge into studying the interaction between organoarsenic species and hybrid nanomaterials in the natural environment.

## 1. Introduction

Water contaminated with arsenic (As) presents a pressing and formidable challenge due to its high toxicity, bioaccumulation potential, and carcinogenic properties [1]. Long-term exposure to wastewater containing arsenic can lead to a range of diseases, including neurological, dermatological, and endocrine disorders [2]. The detrimental effects of arsenic contamination may take over a decade to become apparent, especially at low exposure levels. Therefore, when arsenic contamination arises in water, it has the potential to enter the human body via the food chain or biogeochemical cycle, posing a severe threat to human health [3].

The existence of arsenic in nature displays two forms: organic arsenic and inorganic arsenic. Organic arsenic compounds contain aromatic organoarsenicals and diverse methylated arsenic. In contrast, inorganic arsenic comprises arsenate species (As(V)) and arsenite species (As(III)). Notably, aromatic organoarsenicals, such as p-arsanilic acid (p-ASA) and roxarsone (ROX), have been widely employed in agriculture for decades [4]. They serve the purpose of promoting livestock growth and preventing the proliferation of parasites. These organoarsenicals show limited adsorption and conversion within animals and are primarily excreted through metabolic processes [5]. However, it is crucial to note that organoarsenicals display minimal toxicity and undergo decomposition in soil. They can eventually undergo biotransformation processes, leading to their conversion into various more mobile and toxic inorganic arsenic compounds [6]. In the southern part of China’s Pearl River Delta, p-ASA was detected at 12 μg/kg in the surface soil surrounding pig farms, indicating increased arsenic levels compared to the local background [7]. Additionally, chicken and pig manure in China contained total arsenic ranging from 8.10 × 10^5^ to 5.7 × 10^6^ and from 0.9 × 10^5^ to 2.5 × 10^7^ kg per year [8]. Long-term exposure to arsenic-contaminated drinking water may result in endemic arsenicosis and fatal cancers [9]. Therefore, it is crucial and urgent to remove organic arsenic-contaminated water from the source before its conversion to highly toxic inorganic arsenic in order to prevent arsenic migration and control environmental risks.

Currently, the use of organoarsenicals in agriculture and livestock farming has had a significant environmental impact. The removal of p-ASA and ROX from the environment has become a focal point of research in the environmental field. Disappointingly, research on the removal of organoarsenicals is relatively scarce compared to that on inorganic arsenic, and the development of suitable removal methods is crucial for controlling organic arsenic pollution in livestock [10]. At present, several removal techniques have been proposed, including photodecomposition [5], biodegradation [11], and adsorption [11,12]. Among these methods, adsorption shows the advantages of high efficiency and low cost [13]. Various natural and synthetic adsorbents have been studied for arsenic sorption from water bodies [14]. As a consequence, the development of low-cost, high-efficiency adsorbents has become a research hotspot.

Iron-based adsorbents can form exosphere and endosphere complexes with As(V) owing to their abundant hydroxyl functional groups [15]. Additionally, iron oxides present special characteristics of microporosity distribution and surface charging, facilitating the adsorption of organoarsenicals, while preventing other ions or compounds in the sewage water from interfering with the adsorbent [16]. Consequently, iron-based materials have displayed a wide application in arsenic removal. Moreover, previous research has indicated that binary metal oxides and bimetallic oxides display higher adsorption capabilities compared to pure monometallic iron-based oxides [17]. MnFe_2_O_4_, for instance, not only boasts strong magnetic properties, facilitating separation from solution and reducing the risk of secondary environmental pollution, but also displays better biocompatibility and lower biotoxicity [18]. The low manufacturing cost and simple preparation process of MnFe_2_O_4_ have garnered significant attention. As a result, MnFe_2_O_4_ can be considered a suitable alternative to iron-based materials. However, single MnFe_2_O_4_ suffers from limitations, such as restricted adsorption capacity, slow adsorption rates due to small surface areas, aggregation of active sites, and low overall adsorption efficiency, thus hindering its practical applications [19]. Graphene, a novel two-dimensional carbon material, possesses numerous outstanding properties, including a large specific surface area, strong antimicrobial characteristics, and excellent electrical and mechanical properties. While graphene can serve as an effective adsorbent for pollutants, the challenge lies in efficiently removing it from water after the treatment process [20]. To overcome this problem, an innovative technique that has received much attention is the utilization of magnetic materials for phase separation in aqueous solutions by applying a magnetic field, providing an attractive and cost-effective method for practical operation. Efforts have been made to integrate graphene with magnetic nanoparticles, and these hybridized materials can display enhanced adsorption capabilities [21].

Based on the aforementioned analysis, this research aimed to construct a MnFe_2_O_4_/graphene (rGO) hybrid nanocomposite by employing a facile solvothermal method. And p-ASA and ROX were selected as the typical organoarsenicals to study the adsorption behavior. At the same time, the influence of natural environmental factors on its adsorption efficiency was explored, including ion strength, anion, humus, etc. Finally, a possible adsorption mechanism is proposed. MnFe_2_O_4_/graphene-hybridized nanocomposites show better adsorption properties for organic arsenic compared to the already reported iron-based materials, MWCNT, ZIF-8, etc. (Supplementary Table S1). This research work provides new insights for the preparation and application of new adsorbents.

## 2. Materials and Methods

### 2.1. Reagents and Materials

Graphite was purchased from the Institute of Guangfu Chemical (Tianjin, China), and we also purchased p-arsanilic acid (Aladdin Scientific, Shanghai, China, 98%), roxarsone (Alfa Aesar, Haverhill, MA, USA, 99%), and humic acid (Sigma Aldrich, St. Louis, MO, USA). Ferric chloride hexahydrate (FeCl_3_•6H_2_O), manganese dichloride tetrahydrate (MnCl_2_•4H_2_O), ethylene glycol, sodium acetate anhydrous (NaAc), potassium permanganate (KMnO_4_), hydrogen peroxide (H_2_O_2_, 30%), concentrated sulfuric acid (H_2_SO_4_, 98%), and all other chemicals were analytical reagent grade and purchased from Sinopharm Chemical Reagent Co., Ltd. (Shanghai, China), and used without further purification.

### 2.2. Preparation of MnFe_2_O_4_/rGO Hybrid Nanocomposite

**Scheme 1:**
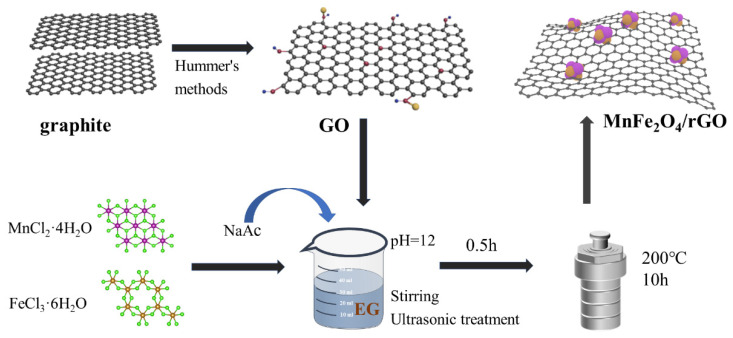
Schematic illustration of the formation process for MnFe_2_O_4_/rGO hybrid nanocomposite.

The graphene (rGO) was synthesized from natural graphite powder by employing a modified Hummers method. The synthesis of MnFe_2_O_4_/rGO was based on a facile one-pot solvothermal method, using FeCl_3_•6H_2_O and MnCl_2_•4H_2_O as starting materials. As depicted in Scheme 1, 0.2 g of GO, 1 g of FeCl_3_•6H_2_O, and 0.376 g of MnCl_2_•4H_2_O were dispersed in 30 mL of ethylene glycol with ultrasonication for 3 h. Later, 3 g of NaAc was added, followed by stirring for 30 min. The mixture was then transferred into a 50 mL Teflon-lined stainless-steel autoclave and heated at 200 °C for 10 h. Solid black product was obtained and washed several times with deionized water and ethanol and vacuum freeze-dried. Bare MnFe_2_O_4_ nanoparticles were also synthesized via a similar approach but in the absence of GO. Also, barely reduced grapheme oxide denoted G was prepared under the same hydrothermal conditions but without MnFe_2_O_4_ nanoparticles.

### 2.3. Characterization

The X-ray diffractograms (XRDs) of the catalysts were analyzed using a Shimadzu XRD-6100 X-ray diffractometer (Kyoto, MA, Japan) with Cu-Kα rays as the X-ray emission source, and the scan range was 5–80°, with a scan rate of 5° min^−1^. Nitrogen adsorption–desorption experiments of the samples were performed on a TriStar II 3020 specific surface and porosity analyzer (Atlanta, MA, USA), and the specific surface area of the samples was calculated using Brunauer–Emmett–Teller (BET) simulations. Transmission electron microscopy (TEM) was used to observe the microstructure of the materials. The samples were dispersed in anhydrous ethanol before testing, 1~2 drops of the suspension were placed on the carbon film, and the carbon film was fixed after the ethanol evaporated and was tested. The infrared spectra of the samples were obtained via an analysis with a Nicolet iS50 infrared spectrometer (Madison, MA, USA) with KBr as the background, scanning wavelengths of 4000–400 cm^−1^, and spectral resolution of 4 cm^−1^. X-ray photoelectron spectroscopy (XPS) analysis was performed by using an ESCALAB 250Xi from Thermo Scientific (Waltham, MA, USA), using XPSPEAK version 41 software for background deduction and peak splitting.

### 2.4. Adsorption Study

In this adsorption experiment, 20 mL of p-ASA and ROX, each with concentrations of 50 mg/L, were individually prepared. Subsequently, 20 mg of MnFe_2_O_4_/rGO adsorbent was added to each solution. The two solutions were subjected to agitation in a shaker at 160 revolutions per minute for varying durations: 1, 3, 5, 10, 30, 60, 180, 240, 720, and 1440 min. Samples were collected, allowed to stand, passed through a membrane, and analyzed. On an Agilent 1200 HPLC (Santa Clara, MA, USA) equipped with a C18 reversed-phase column with a detection wavelength of 264 nm, the mobile phases were water and acetonitrile, the flow rate was 1 mL min^−1^, and the injection volume was 10 L; the concentrations of p-ASA and ROX were determined. When analyzing the concentration of p-ASA, the water: acetonitrile ratio was 90:10; however, when analyzing the concentration of ROX, the ratio was 80:20. Experiments on the thermodynamics of adsorption were conducted at concentrations of 10, 25, 50, 75, and 100, 150, and 200 mg/L, with the temperature of the shaker set at 15, 25, and 35 °C, respectively. The adsorption equilibrium may be determined using Formula (1):

(1) $${\;q}_{e}=\left(C_{0}\;-\;C_{e}\right)\frac{V}{m}$$

where C_0_ is the initial concentration of p-ASA or ROX (mg L^−1^), Ce is the concentration of p-ASA or ROX when it reaches adsorption equilibrium (mg L^−1^), V is the volume of solution added (L), and m is the mass of catalyst (g).

Different ionic intensities were adjusted to 0.01 M, 0.005 M, and 0.001 M with NaNO3 concentrations, and 0.1 M HNO_3_ or 0.1 M NaOH solution was adjusted to 3, 4, 5, 6, 7, 8, 9, and 10; different anion interference experiments were added to 0.01 M NaNO_3_, NaCl, Na_2_SO_4_, and Na_3_PO_4_, respectively; and the pH value of the solution was adjusted to 3, 4, 5, 6, and 7 with 0.1 M HNO_3_ or 0.1 M NaOH solution. A certain concentration of HA (0, 5, 20, and 100 mg/L) was added to the humic acid interference experiment; the pH value of the solution was adjusted to 3, 4, 5, 6, 7, 8, 9, and 10 with 0.1 M HNO_3_ or 0.1 M NaOH solution; and the shaker was shaken for 1440 min at a shaker speed of 160 r/min, sampled, left standing, passed through the membrane, and left standing for analysis.

## 3. Results and Discussion

### 3.1. Properties of MnFe_2_O_4_/rGO Hybrid Nanocomposite

**Figure 1:**
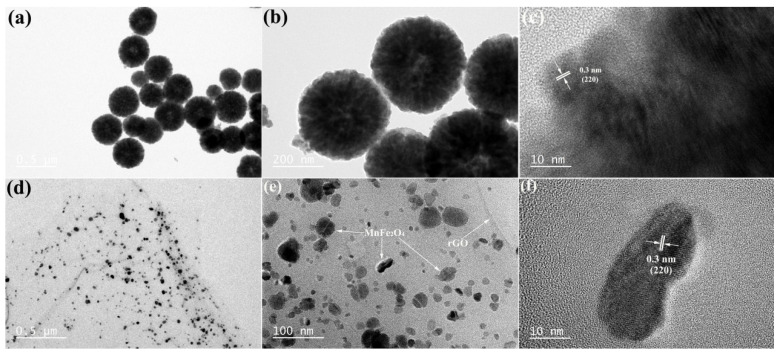
TEM images and high-resolution TEM images of MnFe_2_O_4_ (**a**–**c**) and MnFe_2_O_4_/rGO (**d**–**f**).

Figure 1a,b reveal that monomeric MnFe_2_O_4_ nanoparticles exhibit a circular appearance and form an aggregated microsphere distribution. Additionally, the MnFe_2_O_4_ microspheres are densely packed, creating porous microspheres composed of relatively large nanoparticles with average cluster size diameters ranging from 200 to 400 nm. In contrast, Figure 1d depicts a transmission electron microscopy image of the synthesized MnFe_2_O_4_/rGO, where it is evident that the MnFe_2_O_4_ microspheres are uniformly immobilized on transparent folded graphene sheets without significant clustering. Importantly, a large number of MnFe_2_O_4_ particles remain tightly bound to rGO despite ultrasonication during the preparation of TEM samples, indicating the mechanical stability of the material, which implied that the graphene structure facilitates the prevention of microsphere agglomeration and the formation of strong forces between rGO and MnFe_2_O_4_ [22]. These findings were consistent with those reported in the literature (Figure 1b) [23]. Furthermore, high-resolution transmission electron microscopy (HRTEM) images of both the MnFe_2_O_4_ and MnFe_2_O_4_/rGO composites reveal that the stripes with an interfacial distance of 0.3 nm correspond well to MnFe_2_O_4_ (220) (Figure 1c,f), confirming the successful synthesis of our composite materials.

**Figure 2:**
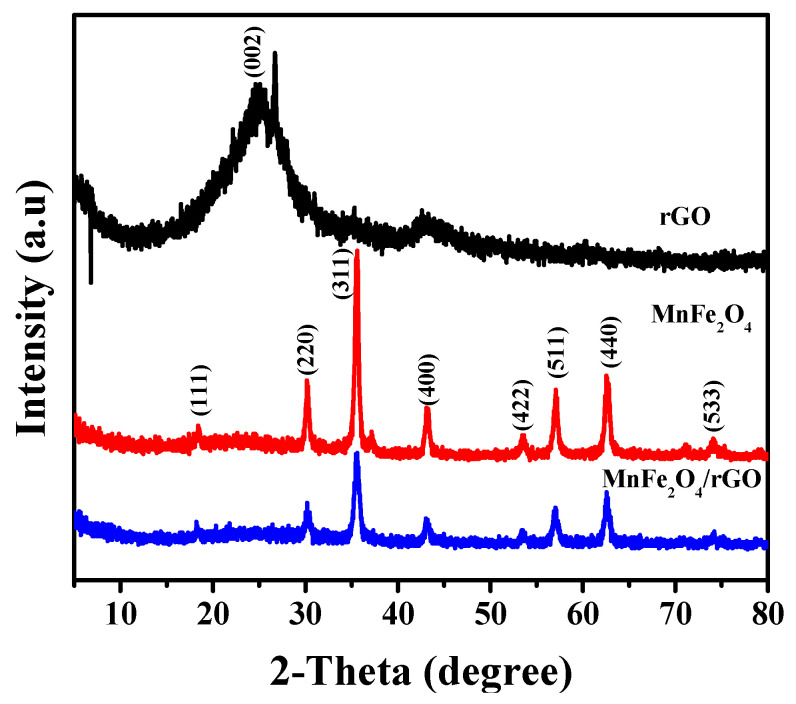
XRD patterns of rGO, MnFe_2_O_4_, and MnFe_2_O_4_/rGO.

The composed MnFe_2_O_4_ nanoparticles were characterized by XRD, as shown in Figure 2. The XRD patterns of MnFe_2_O_4_ nanoparticles could well match the diffraction peaks of cubic spinel-type MnFe_2_O_4_ (JCPDS card No. 10-0319, space group: Fd3m, a = 8.50 Å) [24]. In comparison with the existing literature, rGO shows the absence of the 9.7° peak associated with the GO (001) crystal plane. This absence is attributed to the removal of oxygen-containing functional groups situated between the GO layers during the solvothermal treatment, triggering a reduction reaction that narrows the D spacing according to Bragg’s law. As a result, the interlayer spacing undergoes a significant reduction, closely resembling that of rGO [25]. The characteristic diffraction peak at 2θ = 24.5° (002) typically observed in rGO is conspicuously absent in the as-prepared adsorbent. This absence may be attributed to the infiltration of MnFe_2_O_4_ nanoparticles into the reduced GO layer, causing their separation [26]. Furthermore, the diffraction peaks of MnFe_2_O_4_ crystals closely match those of MnFe_2_O_4_/rGO, indicating that the material is adequately loaded, and the diffraction peak is well-defined and sharpened. The emergence of the strongest diffraction peak of MnFe_2_O_4_ particles at 2θ = 35.86° further validates the formation of nanoparticles and their robust crystallization and growth along the crystal plane (311) [27]. The average grain size, calculated using the Scherrer formula based on the strongest peak (311), is approximately 29.7 nm.

**Figure 3:**
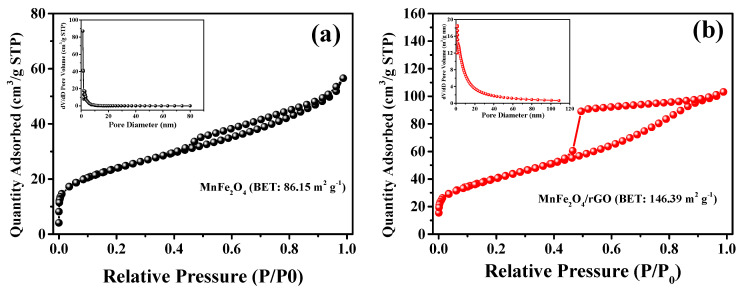
Nitrogen adsorption−desorption isotherms and pore diameter distribution of MnFe_2_O_4_ (**a**) and MnFe_2_O_4_/rGO (**b**).

A nitrogen adsorption–desorption analysis was employed to evaluate the specific surface area and pore size distribution of both monomeric MnFe_2_O_4_ and MnFe_2_O_4_/rGO composites (Figure 3a,b). Utilizing the BET method for nitrogen adsorption and desorption data, the specific surface areas of MnFe_2_O_4_ and MnFe_2_O_4_/rGO nanocomposites were determined to be 86.15 and 146.39 m^2^ g^−1^, respectively. This improvement can be attributed to the uniform distribution of MnFe_2_O_4_ nanoparticles on the rGO sheet. According to the IUPAC classification, the nitrogen adsorption–desorption isotherms and textural properties of MnFe_2_O_4_ nanoparticles exhibit a type IV hysteresis loop, which is characteristic of typical mesoporous materials [28]. In contrast, MnFe_2_O_4_/rGO displays a type IV H3 hysteresis loop [27], indicating the prevalence of mesoporosity and the interconnection between disordered mesoporosity and interparticle mesoporosity. It is well established that the large specific surface area and abundant mesoporous structure of the adsorbent are essential factors contributing to the improved adsorption capacity of the material for reactants [29].

### 3.2. Adsorption Kinetic and Isotherms

**Figure 4:**
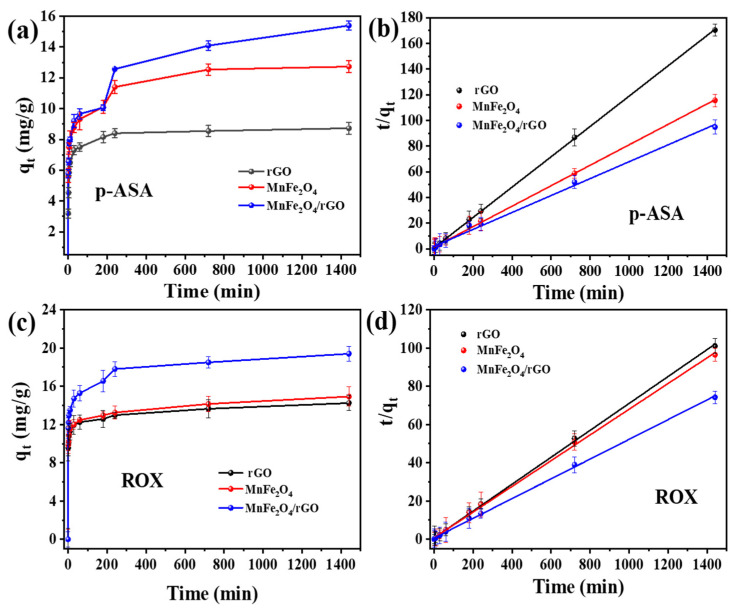
Adsorption performance of various samples for p-ASA (**a**) and ROX (**c**) removal. The dynamic simulation for p-ASA and ROX adsorption onto various samples: (**b**,**d**) pseudo-second-order model.

Kinetic experiments were conducted to analyze the adsorption properties of the prepared materials for arsenic in water. Figure 4a,c illustrate the examination of adsorption kinetics for various organic arsenic species on MnFe_2_O_4_ particles. Active adsorption was observed for both p-ASA and ROX within the initial 100 min of the experiment. Specifically, the MnFe_2_O_4_/rGO complex achieved the removal of 58% of the maximum adsorption capacity for p-ASA and 87% of the maximum adsorption capacity for ROX. Subsequently, between 180 and 300 min, the process continued at a relatively slow rate until equilibrium was reached around 1400 min. In addition, the adsorption rate of the MnFe_2_O_4_/rGO complex was positively correlated with the adsorption rate of rGO and MnFe_2_O_4_. This is due to the fact that MnFe_2_O_4_ loading onto rGO does not agglomerate, and it is also due to the intrinsic adsorption capacity of graphene itself; the combination of the two does not impair their properties, thus elucidating the enhanced adsorption capacity of the complex [30]. Notably, the adsorption of ROX by rGO was more significant. This was perhaps attributed to the presence of more nitroarsenic adsorption sites than aminoarsenic in the adsorption of contaminants by rGO. The presence of vacancies and small pores on the surface of rGO, which can provide additional adsorption sites for nitroaromatic compounds, also resulted in a substantially larger adsorption of ROX by MnFe_2_O_4_/rGO [31].

To confirm the evolving behavior of the adsorbent over the course of the adsorption process, we utilized a pseudo-second-order equation (Equation (2)) to characterize the adsorption of organic arsine on both the monomer MnFe_2_O_4_ and the composite adsorbent.

(2) $$\;\frac{t}{q_{t}}=\frac{1}{{\mathrm{kq}}_{e}^{2}}+\frac{1}{q_{e}}t$$

where k (g mg^−1^ min^−1^) is the rate constant of adsorption, and q_t_ (mg g^−1^) and q_e_ (mg g^−1^) are the adsorption capacity at any time and at equilibrium. The initial sorption rate, h, can be classified as shown in Equation (3):

(3) $$h=kq_{e}^{2}(t\to 0)\;$$

The h (mg g^−1^ min^−1^) and k (g mg^−1^ min^−1^) values can be obtained from the slope and intercept of the t/q_t_ curve. The h and k were calculated for the adsorption of arsenic by MnFe_2_O_4_ in accordance with the fitted secondary kinetic model (R^2^ > 0.99). Based on the h and k values, it was shown that the order of the initial adsorption rates of both adsorbed p-ASA and ROX obeyed rGO < MnFe_2_O_4_ < MnFe_2_O_4_/rGO.

**Figure 5:**
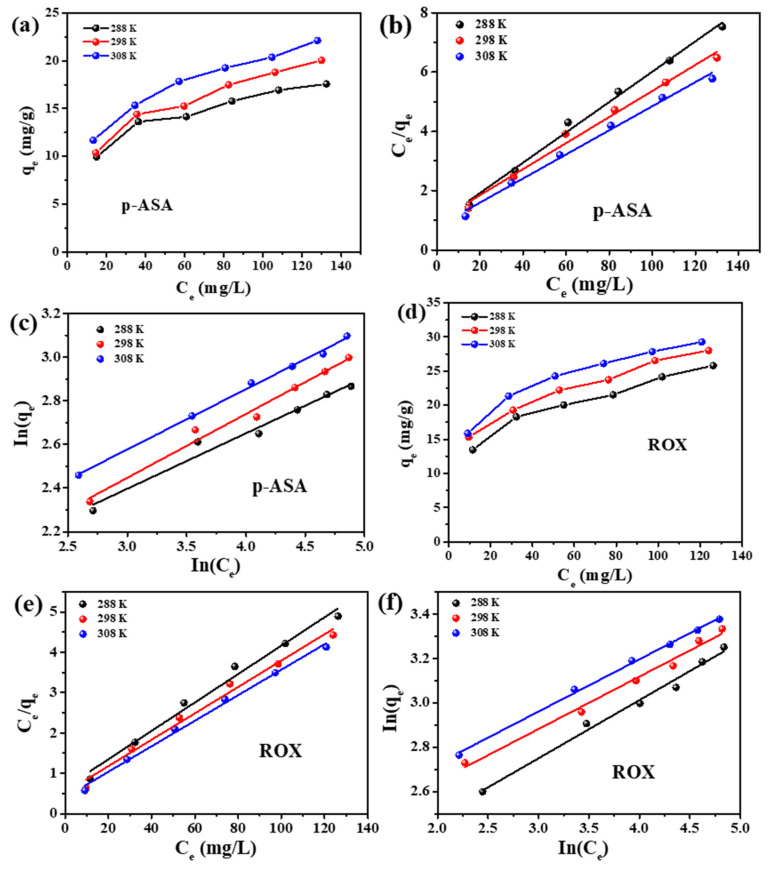
Adsorption isotherms for p-ASA (**a**) and ROX (**d**) adsorption on MnFe_2_O_4_/rGO hybrid nanocomposite, Langmuir fit (**b**,**e**), and Freundlich fit (**c**,**f**).

The adsorption isotherm model elucidates the interaction between adsorbate and adsorbent under equilibrium conditions with a constant pH value. In an equilibrium state characterized by a constant pH value, the adsorption isotherm model is employed to characterize the interaction between the adsorbate and the adsorbent. The analysis of adsorption isotherm data aimed to evaluate the impact of temperature on the adsorption of diverse organic arsenic loads onto MnFe_2_O_4_/rGO and to estimate the maximum adsorption capacity of the adsorbent. The depicted adsorption isotherms of ROX and p-ASA on MnFe_2_O_4_/rGO at three different temperatures are shown in Figure 5a,d. Under the same adsorption conditions, the adsorption capacities of ROX and p-ASA are in the following order: 308K > 298K > 288K. With the rising temperature, the equilibrium absorption increases, suggesting the endothermic character of the adsorption process. At the highest temperature (308K), the maximum adsorption capacities for ROX and p-ASA were 22.4 mg/g and 29.3 mg/g, respectively, indicating a relative advantage in the adsorption of ROX.

The equilibrium adsorption isotherm data were analyzed using the Langmuir and Freundlich isotherm models, and the following are the respective Equations (4) and (5).

(4) $$\mathrm{Langmuir}\;\mathrm{equation}:\;q_{e}=\frac{K_{\mathrm{ad}}q_{\max}C_{e}}{1+K_{\mathrm{ad}}C_{e}}$$

(5) $$\mathrm{Freundlich}\;\mathrm{equation}:\;q_{e}={\;k}_{F}C_{e}^{1/n}\;$$

In the presented equations, K_L_ (mg g^−1^) represents the Langmuir adsorption equilibrium constant, q_m_ stands for the maximum adsorption capacity at monolayer coverage (mg g^−1^), and q_e_ represents the amount of p-ASA and ROX adsorbed at equilibrium (mg g^−1^). Additionally, K_F_ ((mg g^−1^) (L mg^−1^)^1/n^) and n are the Freundlich characteristic constants. To assess the goodness of fit and the degree of error for the validation of the isotherm model, the regression coefficient (R^2^) is employed.

The Langmuir isotherm model assumes that the adsorbate forms a monolayer on the adsorbent surface, and the resulting equation describes the equilibrium between the two phases. The Freundlich isotherm model is employed based on the multilayer adsorption of adsorbates on heterogeneous surfaces. Parameters pertinent to the calculated isotherms are provided in Table S2, and the fitting curves for the expected isotherm models at the three temperatures are depicted in Figure 5b,c,e,f. Both the Langmuir and Freundlich adsorption isotherm models effectively explain the adsorption behavior of organic arsenic during the production of magnetic adsorbents, as indicated by the R^2^, all of which exceed 0.97.

By employing the Langmuir dimensionless constant separation factor (RL) [23], we can theoretically demonstrate the favorable nature of the adsorption process for organic arsenic and MnFe_2_O_4_/rGO. RL values greater than 1 suggest an unfavorable type of isotherm, while values between 0 and 1 indicate a favorable type, as expressed in Equation (6) below.

(6) $$R_{L}=\frac{1}{1+K_{L}C_{e}}$$

where C_e_ is the equilibrium concentration of adsorbate (mg L^−1^), and K_L_ is the Langmuir constant. The calculated values are shown in Table S3.

The adsorption of organic arsenic onto the MnFe_2_O_4_/rGO composite was shown to be temperature-independently advantageous, with RL values ranging from 0.686 to 0.767 (0 < R_L_ < 1).

According to the calculation of Langmuir adsorption isotherm, the maximum adsorption capacity of p-ASA and ROX on MnFe_2_O_4_/rGO was 24.6 mg g^−1^ and 31.62 mg g^−1^, respectively, and the maximum adsorption capacity of p-ASA and ROX at 298 K was 22.75 mg g^−1^ and 30.59 mg g^−1^, respectively.

### 3.3. Effect of pH and Ionic Strength

**Figure 6:**
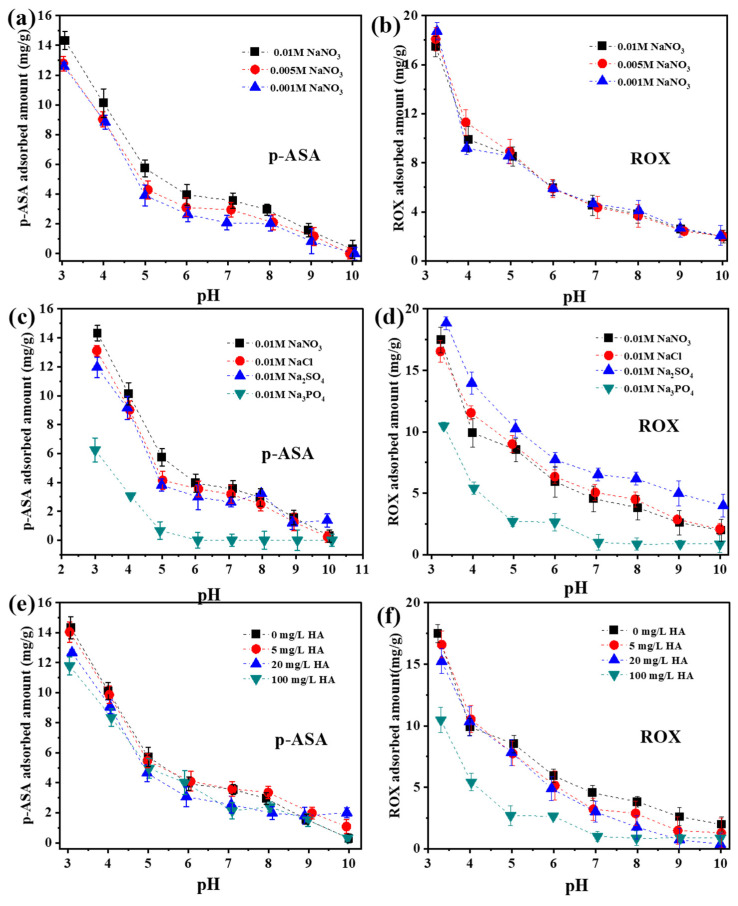
Effect of ionic strength (**a**,**b**), different anions (**c**,**d**), and humic acid (**e**,**f**) on the adsorption of p-ASA and ROX onto MnFe_2_O_4_/rGO hybrid nanocomposite.

Natural aqueous environmental media typically contain a variety of different compounds, which can potentially interfere with the adsorption process of target ions. These ions may either enhance or hinder the adsorption of organic arsenicals. Therefore, investigating the impact of ionic strength and other ions on the adsorption process can provide valuable insights into the mechanisms of adsorption. Initially, the effect of varying the concentration of ${NO}_{3}^{-}$ (0.01 M, 0.005 M, and 0.001 M) was investigated, and it is observed from Figure 6a,b that the ionic strength variation has limited and almost no effect on the adsorption of p-ASA and ROX, thus suggesting that the organic arsenicals form an inner sphere complex on the MnFe_2_O_4_/rGO surface, thereby avoiding competition with other ions [32].

### 3.4. Effect of Background Anions

Competition with naturally occurring anions in aquatic environments can affect the adsorption of arsenic. Therefore, four common anions (Cl^−^, ${NO}_{3}^{-}$, ${SO}_{4}^{2-}$, and ${PO}_{4}^{3-}$) were chosen to investigate their influence on the MnFe_2_O_4_/rGO adsorption process [32]. Intriguingly, 0.01 M concentrations of Cl^−^, ${NO}_{3}^{-}$, and ${SO}_{4}^{2-}$ ions at different pH levels had minimal effects on p-ASA and ROX adsorption. The removal efficiency of the two organic arsenic compounds remained at approximately 90% of their initial rates. The ${PO}_{4}^{3-}$ anions had a significant impact on the adsorption of p-ASA and ROX, leading to a reduction in the removal of organic arsenic by 50–75%. Phosphorus and arsenic are both nonmetals belonging to the VA group and share similar chemical characteristics. It has been reported that both phosphate and arsenate are tetrahedral anions, and phosphate can potentially form inner-sphere complexes with hydroxyl groups on the adsorbent surface, unlike other ions [33,34]. Similar results have been reported in numerous studies, indicating that the presence of phosphate effectively reduces the adsorption of arsenic compounds by metal oxides [35,36]. Regarding Cl^−^, ${NO}_{3}^{-}$, and ${SO}_{4}^{2-}$, which have a minor inhibitory effect on adsorption, this is attributed to diffusion-based outer-sphere complexation adsorption. This suggests that the absence of electrostatic interactions has a minimal effect on the adsorption capacity.

### 3.5. Effect of HA

Groundwater systems with elevated arsenic levels are distinguished by a substantial concentration of dissolved organic matter. This organic matter governs the transport and transformation of contaminants within these systems, playing a pivotal role in the release of pollutants. In this context, we employed ROX and p-ASA as representative models of organic arsenicals. These compounds were subjected to adsorption studies in the presence of HA at varying concentrations of 0, 5, 20, and 100 mg·L^−1^. As illustrated in Figure 6e,f, the adsorption of organic arsenic was impeded under the presence of p-ASA, particularly at pH values below 6, in a manner proportionate to the concentration of HA. However, a more comparable adsorption pattern emerged at pH levels exceeding 6. In contrast, the adsorption of ROX was progressively suppressed across the entire pH range with the increasing humic acid concentration. Distinct concentrations of humic acid similarly imposed inhibitory effects on the adsorption of organic arsenic, particularly at a lower pH level.

**Figure 7:**
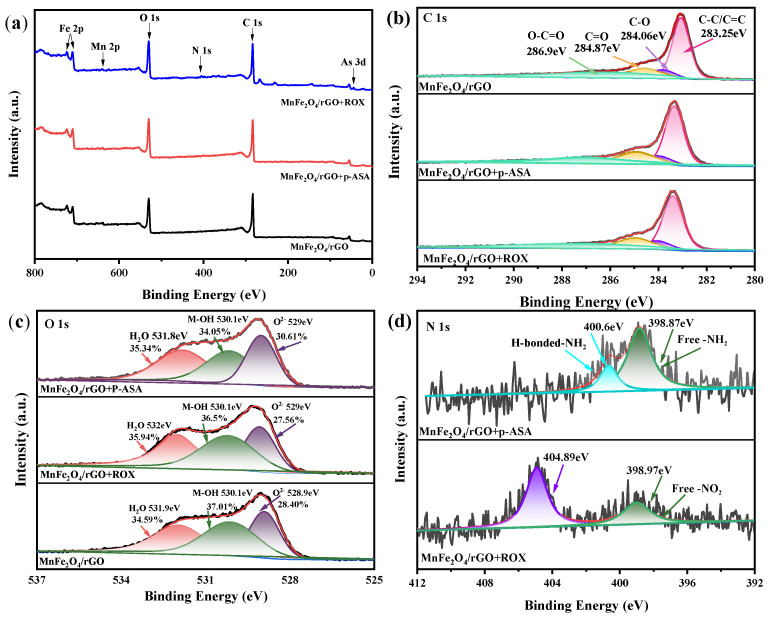
XPS spectra of MnFe_2_O_4_/rGO hybrid nanocomposite before and after organoarsenic adsorption: (**a**) survey, (**b**) C 1s, (**c**) O 1s, and (**d**) N 1s.

The elemental composition and valence states of MnFe_2_O_4_/rGO were analyzed both before and after adsorption, using XPS. In Figure 7a, the full spectrum of arsenic species is depicted before and after adsorption, with the presence of elements C, O, N, Fe, and Mn observed in all samples. Figure 7b displays the characteristic peaks of C1s. This high-resolution C1s spectrum can be deconvoluted into four peaks situated at 283.16 eV, 283.6 eV, 284.67 eV, and 287 eV, corresponding to the binding energies of C-C/C=C, C-O, C=O, and O-C=O bonds [37]. It is worth noting that the percentage content of oxygen-containing functional groups is lower compared to that reported in the literature [38]. Additionally, this finding reinforces the conclusion regarding the rGO during XRD analysis. It suggests that the conjugated π-orbital structure of graphite is disrupted, and oxygen-containing functional groups are interspersed within the graphene structure during the fabrication process [39].

A multi-peak Gaussian fit of MnFe_2_O_4_/rGO before the adsorption of organic arsenic was performed to deconvolve the O 1s (Figure 7c) spectrum into three peaks centered at 528.9 eV, 530.1 eV, and 534.5 eV. The main contributions are the lattice oxygen in MnFe_2_O_4_ [40], the surface metal hydroxyl group (M-OH) from the oxygen-containing functional group of rGO [41], and the surface adsorbed water molecules. Significantly, the MnFe_2_O_4_/rGO surface had a high percentage of M-OH (37.01%), which is considered to be an important component for the removal of contaminants [42]. Our experimental observations are also as expected. After adsorption of p-ASA and ROX, the oxygen-containing functional groups on the surface are less than 34.05% and 32.52%, while the O^2−^ on the surface increases from 28.40% to 30.61% and 31.50%, respectively, which may be attributed to the specific adsorption of the lattice oxygen by various arsenic species. There was a slight magnitude in the variation in the surface adsorption percentage of H_2_O in MnFe_2_O_4_/rGO prior to and following adsorption, omitting the contribution of H_2_O to removal. In addition, the fact that the percentage content of M-OH is lower in the ROX-loaded MnFe_2_O_4_/rGO in comparison to the adsorbed p-ASA indirectly means that the adsorbent facilitates the removal of ROX contaminants. In addition to electrostatic mutual interaction and surface complexation for adsorption, hydrogen bonding serves as another important principle used to explain the removal of ROX in liquid-phase adsorption. Moreover, MnFe_2_O_4_/rGO, p-ASA, and ROX in this study all have sufficient hydroxyl (-OH), amino (-NH_2_), and nitro (-NO_2_) to provide H-donors or H-acceptors. As shown in Figure 7d, the observation of N1s revealed the presence of hydrogen bonding after the adsorption of organic matter [43]. Crucially, bonding in hydrogen is also influenced by hydroxyl groups, and the increase in hydroxyl (-OH) groups leads to a monotonic increase in the amount of adsorbed arsenic contaminants [44], and this, combined with the above factors, is more advantageous for the removal of ROX from water.

Supplementary Figure S2 demonstrates the adsorption of p-ASA and ROX onto MnFe_2_O_4_/rGO, with peaks at 46.7 eV and 44.06 eV that are both assigned to AS^V^-O [45]. This is consistent with the analysis from ATR-FTIR measurements, indicating that different forms of arsenic are adsorbed onto MnFe_2_O_4_ by forming As-O-M (M = Fe or Mn) bonds to create surface complexes [46]. Supplementary Figure S3 illustrates the adsorption of organic arsenic on this composite material, showing that the binding energy of Fe2p and Mn2p remains nearly unchanged in the chemical state before and after adsorption. This not only confirms the presence of both Fe and Mn elements in the MnFe_2_O_4_/rGO hybrid material but also suggests that the adsorption process occurs on the surface without involving redox reactions. A slight shift in two of the peaks can be attributed to the interaction with organo-arsenic in MnFe_2_O_4_/rGO.

### 3.6. Adsorption Mechanism

As is well known, the zeta potential of a species is measured as a function of the pH to assess the surface’s acidity or alkalinity and determine the isoelectric point (IEP). This experiment was conducted under the working conditions at pH = 3 (Supplementary Figure S4). Observations suggest that the isoelectric point (IEP) of MnFe_2_O_4_/rGO is approximately pH = 5. Our experimental pH is lower than the protonated surface IEP of MnFe_2_O_4/_rGO, which is positively charged [47]. p-ASA and ROX can form charged species (due to the presence of acidic (nitro) and basic (amino) groups), and their pKa1 values are listed in Supplementary Figure S5. At pH = 3, both p-ASA and ROX exist in the form of molecules and mononegative ions. p-ASA has a lower pKa (1.9), indicating lower solubility, resulting in C_6_H_7_${AsNO}_{3}^{-}$ and making up approximately 30% of the ions in solution. In contrast, ROX has a relatively higher pKa1 (3.43), with C_6_H_5_${AsNO}_{6}^{-}$ constituting approximately 83% of the ions, and monovalent ions dominate the solution. Consequently, the adsorbent can bind organic pollutants to its surface through electrostatic attraction, thereby facilitating their removal. Additionally, the greater the hydrolysis of ROX in an aqueous solution, the higher the corresponding adsorption capacity. Moreover, considering the O fraction peak in XPS and the adsorbent’s behavior in the presence of interference from other ions, we suggest the occurrence of surface complexation. Notably, post-adsorption, iron experiences a substantial shift (Supplementary Figure S3), indicating iron’s involvement in the complexation reaction with organic arsenic within MnFe_2_O_4_/rGO. This leads us to speculate that a monodentate surface complexation reaction occurs with organic arsenic, resulting in a reduction in the surface hydroxyl content of MnFe_2_O_4_/rGO. This observation aligns with the previously described FTIR analysis results regarding the formation of Fe-O-As bonds, as in Equation (7) [31,36]:

Fe−OH_suf_ + AsO_3_R^3−^ + H^+^ = FeAsO^+^_3_R^2−^ + H_2_O (7)

**Figure 8:**
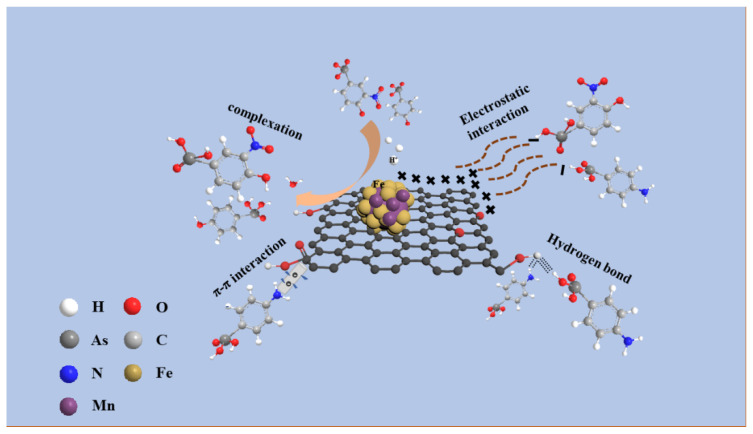
Possible adsorption mechanism of p-ASA and ROX over MnFe_2_O_4_/rGO hybrid nanocomposite.

Another significant mechanism for elucidating liquid-phase adsorption is through the formation of hydrogen bonds. Since both the adsorbent and the adsorbate have hydrogen donor or hydrogen acceptor groups. The abundance of hydroxyl groups in the MnFe_2_O_4_-reduced graphene oxide complex enables the formation of hydrogen bonds with the amino (-NH_2_) or hydroxyl (-OH) groups present in the p-ASA molecule (O-H⋅⋅⋅N, O-H⋅⋅⋅O) [43]. However, in comparison with the prior literature, it was noted that the adsorption of metal oxides on organic arsenic typically results in a higher adsorption capacity for p-ASA than for ROX [19,44,48]. This difference can be attributed to the inclusion of oxygen-containing groups in the reduced graphene oxide composite prepared in this study. Nitroaromatic compounds serve as π-electron acceptors known as π-deficient aromatic molecules (NAC). The presence of oxygen-containing groups/defects/edges on the rGO surface provides electron-donating capabilities, resulting in π-π interactions beyond EDA (electron–donor–acceptor) interactions. The nitro functional groups of NAC not only enhance π-π EDA interactions but also facilitate electrostatic interactions with the oxygen-containing groups/edges/defects present in graphene materials [31] (Figure 8). MnFe_2_O_4_/rGO retains the characteristics of reduced graphene oxide (rGO) and exhibits a heightened affinity for ROX.

## 4. Conclusions

In this work, a MnFe_2_O_4_/rGO hybrid nanocomposite was successfully synthesized via a facile solvothermal method and employed to adsorb two kinds of aromatic organoarsenic. The synergism between MnFe_2_O_4_ and graphene in the MnFe_2_O_4_/rGO nanocomposite enables its high affinity towards p-ASA and ROX. The maximum adsorption capacities of p-ASA and ROX onto MnFe_2_O_4_/rGO were 24.6 and 31.62 mg g^−1^ on account of the Langmuir adsorption isotherm. Additionally, the chemical structure of organoarsenic displays a significant impact on the adsorption capacities. Surface complexation and electrostatic interaction are the primary adsorption mechanisms on the basis of the XPS and FTIR analysis. In general, this research shows the potential value of the MnFe_2_O_4_/rGO hybrid nanocomposite as a recyclable adsorbent in the purification of natural water contaminated with arsenic.

## Data Availability

The data presented in this study are available upon request from the corresponding author.

## Supplementary Materials

The following supporting information can be downloaded at https://www.mdpi.com/article/10.3390/ma16247636/s1. Figure S1: FT-IR spectra of MnFe_2_O_4_ and MnFe_2_O_4_/rGO; Table S1: Comparison of maximum adsorption capacity (*q_max_*) of p-ASA and ROX on adsorbents. Table S2: Calculated equilibrium constants for p-ASA and ROX adsorption onto MnFe_2_O_4_/rGO; Table S3: RL values calculated for all temperatures and concentrations; Figure S2: As 3d XPS spectra of MnFe_2_O_4_/rGO after p-ASA and ROX adsorption; Figure S3: Fe 2p XPS spectra and Mn 2p XPS spectra; Figure S4: pH dependent zeta-potential plots of MnFe_2_O_4_/rGO; Figure S5: The chemical structure formula, acidity constant and existing form in water of p-ASA (a) and ROX (b). References [49,50,51,52,53,54,55,56] are cited in the Supplementary Materials.
