# Supplementary material for: Adsorption Behavior of Organoarsenicals over MnFe2O4-Graphene Hybrid Nanocomposite: The Role of Organoarsenic Chemical Structures
Source: Materials (Basel). 2023 Dec 14;16(24):7636. doi: 10.3390/ma16247636 (PMC10744441; doi:10.3390/ma16247636)
Supplement: Supplementary file 1 [file materials-16-07636-s001.zip › materials-2761893-supplementary.pdf]

Adsorption behavior of organoarsenicals over $MnFe_2O_4$-graphene hybrid nanocomposite: The role of organoarsenic chemical structures

Binxian Gu [1, 2], Haijie Zhang [1], Meng Ye [1], Ting Zhou [1], Jianjian Yi [1], Qingsong Hu [1,]*

[1] College of Environmental Science and Engineering, Yangzhou University, 196 West Huayang Road, Yangzhou, 225127, PR China

[2] Jiangsu Collaborative Innovation Center for Solid Organic Waste Resource Utilization, Nanjing, 210095, PR China

*Corresponding author:

Dr. Qingsong Hu

E-mail address: huqs@yzu.edu.cn

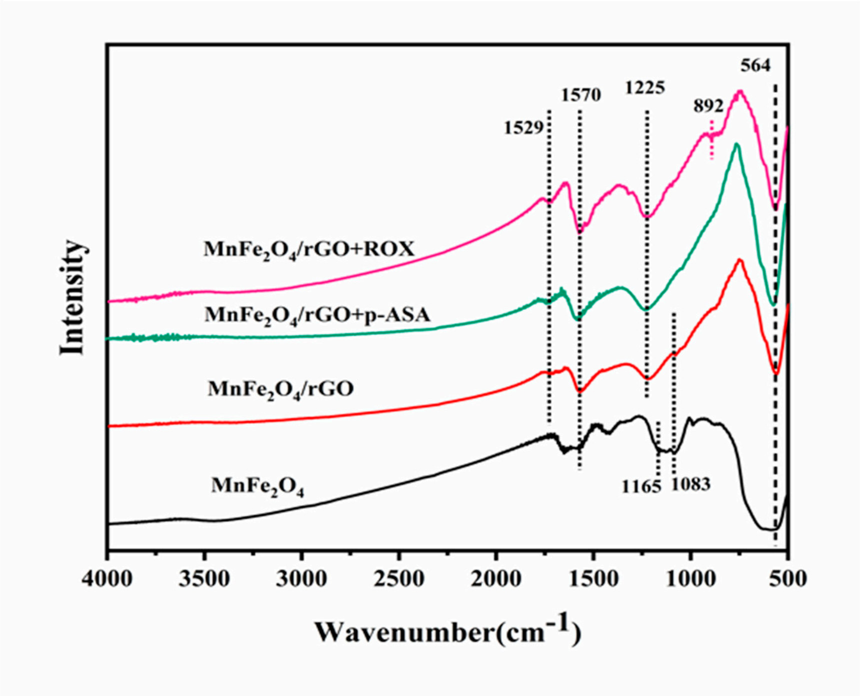

Fig. S1 FT-IR spectra of $MnFe_2O_4$ and $MnFe_2O_4$/rGO

As indicated in Fig. S1, FT-IR analysis was done on the produced nanocomposites both before and after the adsorption of organic arsenic to investigate the variability of the various functional groups of the material. Two peaks at approximately 3300 cm^{-1} and 1570 cm^{-1} in all samples were due to the stretching and vibrations of the OH (which has been smoothed) and H-O-H groups of the water molecule, respectively[1]. The bands at 564 cm^{-1} for $MnFe_2O_4$/rGO, as in the case of the complex samples with adsorbed organic arsenic, correspond to Fe-O stretching vibrations in $MnFe_2O_4$ for all of the samples, whereas at 1529 cm^{-1} only $MnFe_2O_4$ does not appear as a peak, which can be attributed to the aromatic C=C bonding, also confirming the presence of GO in the material[2]. In contrast, the two peaks of $MnFe_2O_4$ at about 1165 cm^{-1} and 1083 cm^{-1} should correspond to the bending vibration mode of the surface metal hydroxyl group (M-OH, M for Fe or Mn)[3]. However, the strength of the peaks corresponding to the $MnFe_2O_4$/rGO complex and

the p-ASA and ROX components that had been adsorbed decreased. During the adsorption phase, the presence of peak shifts suggests the existence of contact forces between hydroxyl groups and organic arsenic. Furthermore, in the adsorption degradation of ROX by $MnFe_2O_4$/rGO, a new peak at 875 cm^{-1} appears corresponding to the As-O stretching vibration[4, 5], revealing the successful adsorption of arsenic contaminants to the surface.

Table S1 Comparison of maximum adsorption capacity (q_{max}) of p-ASA and ROX on adsorbents.

Adsorbents	pH	Adsorption Capacity mmol g^{-1}		Ref.
		p-ASA	ROX	
Goethite	5.0	0.98	0.99	[6]
Activated Carbon	-	1.1	1.5	[7]
MIL-100-Fe	-	1.68	1.8	
ZIF-8	-	3.3	-	[8]
GAC	7.0	-	2.4	[9]
MWCNT	6.0	-	0.049	[10]
FMBO	4.0	0.79	0.51	
FeOOH	4.0	0.45	0.32	[11]
MnO_2	4.0	0.55	0.27	

Values were converted from mg/g

Table S2 Calculated equilibrium constants for p-ASA and ROX adsorption onto $MnFe_2O_4$/rGO.

sample	T (K)	Langmuir			Freundlich		
		K_L (L mg^{-1})	q_{max} (mg g^{-1})	R^2	K_F (L mg^{-1})	n	R^2
p-ASA	288	17.49	19.53	0.992	5.12	3.93	0.966
	298	22.03	22.75	0.986	4.80	3.41	0.979
	308	19.27	24.60	0.990	5.74	3.62	0.986
ROX	288	18.29	28.41	0.982	7.12	3.81	0.971
	298	16.06	30.59	0.983	8.78	4.23	0.971
	308	12.81	31.62	0.994	9.52	4.24	0.987

Table S3 RL values calculated for all temperatures and concentrations.

Temperature	288	298	308	288	298	308
C_0 (mg L^{-1})	RL					
	p-ASA	p-ASA	p-ASA	ROX	ROX	ROX
	0.767	0.694	0.702	0.686	0.690	0.727

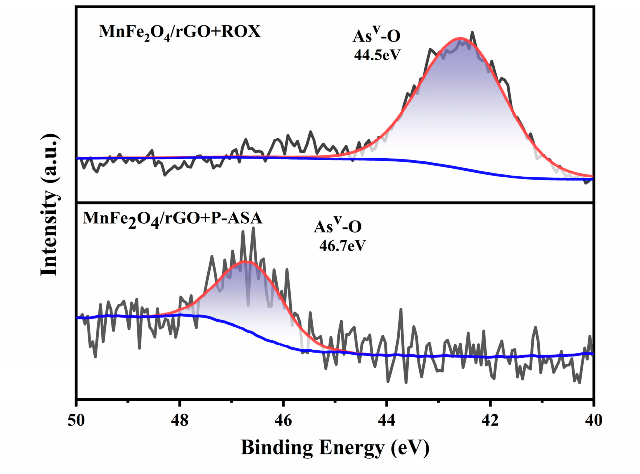

Fig. S2 As 3d XPS spectra of $MnFe_2O_4$/rGO after p-ASA and ROX adsorption.

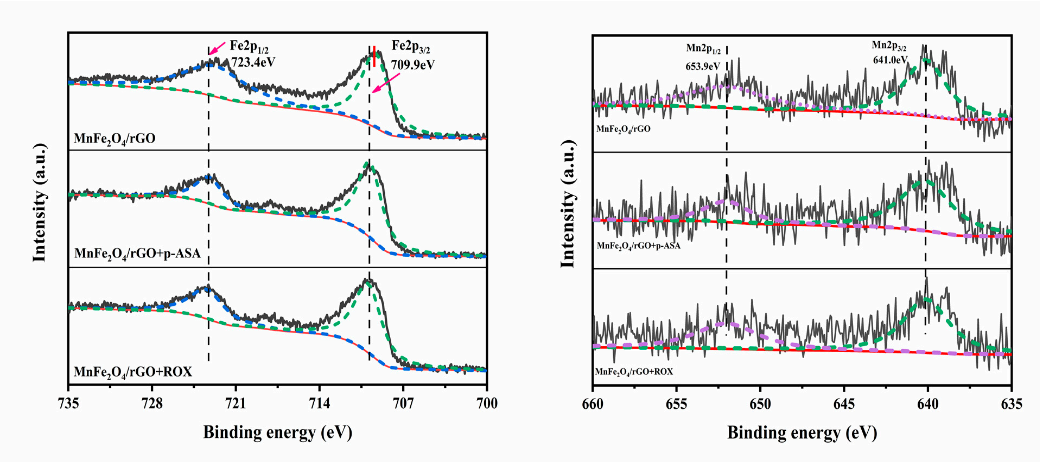

Fig. S3 Fe 2p XPS spectra and Mn 2p XPS spectra

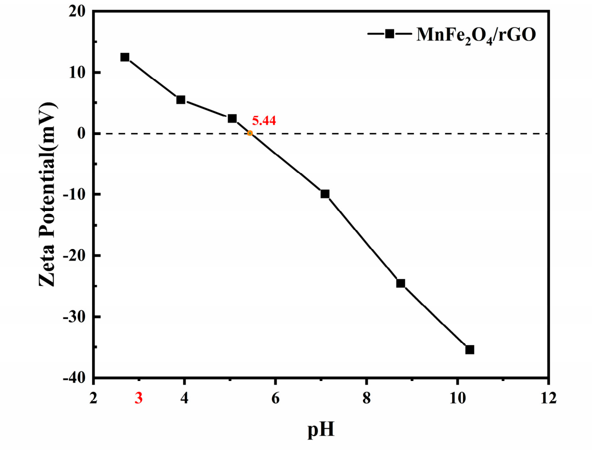

Fig. S4 pH dependent zeta-potential plots of $MnFe_2O_4$/rGO

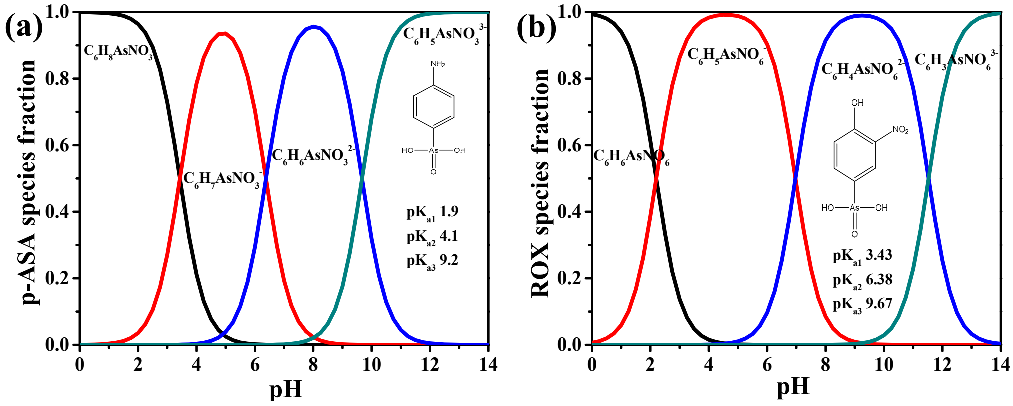

Fig. S5 The chemical structure formula, acidity constant and existing form in water of p-ASA (a) and ROX (b)

References

1. Liu, J.; Ren, S.; Cao, J.; Tsang, D. C. W.; Beiyuan, J.; Peng, Y.; Fang, F.; She, J.; Yin, M.; Shen, N.; Wang, J., Highly efficient removal of thallium in wastewater by $MnFe_2O_4$-biochar composite. *Journal of Hazardous Materials* **2021,** 401, 123311.
2. Al-Gaashani, R.; Najjar, A.; Zakaria, Y.; Mansour, S.; Atieh, M. A., XPS and structural studies of high quality graphene oxide and reduced graphene oxide prepared by different chemical oxidation methods. *Ceramics International* **2019,** 45, (11), 14439-14448.
3. Wen, Z.; Zhang, Y.; Guo, S.; Chen, R., Facile template-free fabrication of iron manganese bimetal oxides nanospheres with excellent capability for heavy metals removal. *Journal of Colloid and Interface Science* **2017,** 486, 211-218.
4. Joshi, T. P.; Zhang, G.; Koju, R.; Qi, Z.; Liu, R.; Liu, H.; Qu, J., The removal efficiency and insight into the mechanism of para arsanilic acid adsorption on Fe-Mn framework. *Science of The Total Environment* **2017,** 601-602, 713-722.
5. Zhang, G.-S.; Qu, J.-H.; Liu, H.-J.; Liu, R.-P.; Li, G.-T., Removal Mechanism of As(III) by a Novel Fe−Mn Binary Oxide Adsorbent: Oxidation and Sorption. *Environmental Science & Technology* **2007,** 41, (13), 4613-4619.
6. Chen, W.-R.; Huang, C.-H., Surface adsorption of organoarsenic roxarsone and arsanilic acid on iron and aluminum oxides. *Journal of Hazardous Materials* **2012,** 227-228, 378-385.
7. Jun, J. W.; Tong, M.; Jung, B. K.; Hasan, Z.; Zhong, C.; Jhung, S. H., Effect of Central Metal Ions of Analogous Metal–Organic Frameworks on Adsorption of Organoarsenic Compounds from Water: Plausible Mechanism of Adsorption and Water Purification. **2015,** 21, (1), 347-354.
8. Jung, B. K.; Jun, J. W.; Hasan, Z.; Jhung, S. H. J. C. E. J., Adsorptive removal of p-arsanilic acid from water using mesoporous zeolitic imidazolate framework-8. **2015,** 267, 9-15.
9. Poon, L.; Younus, S.; Wilson, L., Adsorption study of an organo-arsenical with

chitosan-based sorbents. *Journal of Colloid and Interface Science* **2014,** 420, 136–144.

10. Hu, J.; Tong, Z.; Hu, Z.; Chen, G.; Chen, T., Adsorption of roxarsone from aqueous solution by multi-walled carbon nanotubes. *Journal of Colloid and Interface Science* **2012,** 377, (1), 355-361.

11. Joshi, T. P.; Zhang, G.; Jefferson, W. A.; Perfilev, A. V.; Liu, R.; Liu, H.; Qu, J., Adsorption of aromatic organoarsenic compounds by ferric and manganese binary oxide and description of the associated mechanism. *Chemical Engineering Journal* **2017,** 309, 577-587.
